# Polyneuropathy in Acute Lymphoblastic Leukemia Long-Term Survivors: Clinical and Electrophysiological Characteristics With the Impact of Radiotherapy

**DOI:** 10.3389/fped.2020.526235

**Published:** 2021-02-09

**Authors:** Slawomir Kroczka, Konrad Stepien, Izabela Witek-Motyl, Tomasz Klekawka, Eryk Kapusta, Agnieszka Biedron, Pawel Skorek, Hanna Twardowska, Klaudia Stasik, Szymon Skoczen

**Affiliations:** ^1^Department of Child and Adolescent Neurology, Jagiellonian University Medical College, Krakow, Poland; ^2^Department of Child Neurology, University Children's Hospital, Krakow, Poland; ^3^Department of Oncology and Hematology, University Children's Hospital, Krakow, Poland; ^4^Department of Pediatric Oncology and Hematology, Institute of Pediatrics, Jagiellonian University Medical College, Krakow, Poland; ^5^Student Scientific Group of Pediatric Oncology and Hematology, Jagiellonian University Medical College, Krakow, Poland

**Keywords:** acute lymphoblastic leukemia, survivors, polyneuropathy, electroneurography, electromyography, radiotherapy

## Abstract

**Introduction:** Acute lymphoblastic leukemia (ALL) is the most common childhood cancer with one of the highest survival rates. Long-term complications that occur after intensive oncological treatment often impair normal daily functioning. However, existing data on peripheral nervous system condition in ALL survivors remain conflicting.

**Materials and Methods:** The study group consisted of 215 ALL survivors. Patients were treated with New York (NY, *n* = 45), previous modified Berlin–Frankfurt–Münster (pBFM, *n* = 64), and BFM95 (*n* = 106) protocols. Time elapsed between the end of the treatment and the control examination varied from 0.3 to 20.9 years. The analyzed patients underwent a neurophysiological analysis with electroneurography (ENG) of motor (median and peroneal) and sensory (median and sural) nerves as well as electromyography (EMG) of tibialis anterior, vastus lateralis, and interosseous I muscles. To estimate the influence of radiotherapy on recorded neurophysiological responses, a joint analysis of NY, and pBFM groups was performed.

**Results:** Clinical symptoms of polyneuropathy were noted among 102 (47.4%) children during the ALL therapy and in 111 (51.6%) during follow-up. At the time of treatment, polyneuropathy was diagnosed in 57.8% participants from NY group, 35.9%—pBFM and 50.0%—BFM95 (*p* = 0.145). A significantly higher incidence of polyneuropathy was observed during a follow-up in the NY group (68.9%; *p* < 0.001 vs. pBFM, *p* = 0.002 vs. BFM95). The most frequent abnormality within all the protocols was demyelination (NY: 44.4%, pBFM: 59.4%, BFM95: 41.5%), in contrast to the least frequently registered isolated axonal changes. The negative influence of oncological treatment on neurophysiological parameters in ALL survivors was observed. Complex disorders of motor nerves, sensory nerves, and motor unit potentials were registered. Motor-sensory neuropathy was the most frequently found pathology in all analyzed protocols. The harmful effect of radiotherapy was also observed in EMG results.

**Conclusions:** Detailed neurophysiological analysis in long-term childhood ALL survivors has shown generalized abnormalities in registered parameters. To our knowledge, the current study is the largest and one of the most comprehensive ones among those examining disturbances in ENG and EMG in this group of patients. Moreover, we are the first ones to demonstrate the negative influence of radiotherapy on peripheral nerve conduction parameters.

## Introduction

Acute lymphoblastic leukemia (ALL) is the most common type of cancer in a pediatric population (1). Due to the modern ALL treatment based on chemotherapy, an unexpectedly high survival rate has been observed. Rapidly growing ALL survivors population and potential long-term treatment complications encourage further research and development of effective late-effects screening methods.

Chemotherapy-induced peripheral neuropathy (CIPN) is one of the major problems in pediatric ALL survivors. However, it is still insufficiently investigated. It was reported that acute CIPN is observed in 20–60% of patients treated for childhood malignancies (2). Lavoie Smith et al. showed that CIPN occurs in 78% of ALL patients treated with vincristine (3). On the other hand, a vincristine-induced peripheral neuropathy defined simultaneously by clinical and electrophysiological features was reported by Tay et al. (4) in about 15.8% of ALL long-term survivors. A significant variation in the reported incidence of CIPN is undoubtedly caused by the type of chemotherapy, different dosages as well as poorly investigated genetic factors (5).

Clinical symptoms of CIPN are mostly subtle and commonly unreported, especially in the pediatric population. Up to 40% of sensory and 15% of motor neuropathies were missed (2). For this reason, all patients treated with neurotoxic chemotherapeutics should be routinely screened for CIPN (2). According to the current guidelines, such screening should be based on a complex patient and parents interview with specific questions and precise physical examination. Moreover, an exact functional assessment is sufficient for CIPN diagnosis and treatment decisions. It was suggested that objective neurophysiological testing should be considered as a last diagnostic tool, which is necessary in doubtful and demanding cases (2).

Vincristine, used in modern treatment protocols, is one of the chemotherapeutic agents with proven neurotoxic activity. This microtubule-targeting drug impairs a vesicle-mediated neuron transport and potentially induces axonal degeneration (6). As noted previously, this process is similar to the Wallerian degeneration (6). In addition, the neurotoxic effect of vincristine is also associated with hyperactivity of C fibers which leads to allodynia and degeneration of myelinated A fibers (7).

This phenomenon can be precisely registered with electroneurophysiological methods. However, recent studies have often been inconsistent in neurophysiological evaluation using electroneurography (ENG) and electromyography (EMG) (4, 8–13). One of the characteristic signs of axonal injury is a decrease in the amplitude of the motor and sensory potentials with normal or slightly impaired conduction velocity and concomitant features of acute muscle denervation. Significant decrease of conduction velocity with prolonged latency and delayed F wave are specific to demyelination of peripheral nerves (2).

The aim of our study was to conduct a complex neurophysiological evaluation of the peripheral nervous system in a group of childhood ALL survivors treated with different protocols. In particular, we wanted to determine the impact of radiotherapy on peripheral nerve conduction parameters.

## Materials and Methods

The study group consisted of 215 patients who underwent ALL treatment. The age of the patients ranged between 1 and 18 years with a mean of 5.3 ± 3.5 years at the beginning of treatment and 14.1 ± 5.3 years at follow-up. The time range between the end of treatment and performance of neurophysiological follow-up analyses varied from 0.3 to 20.9 years. The study group consisted of 108 boys (50.2%) and 107 girls (49.8%).

**Figure 1:**
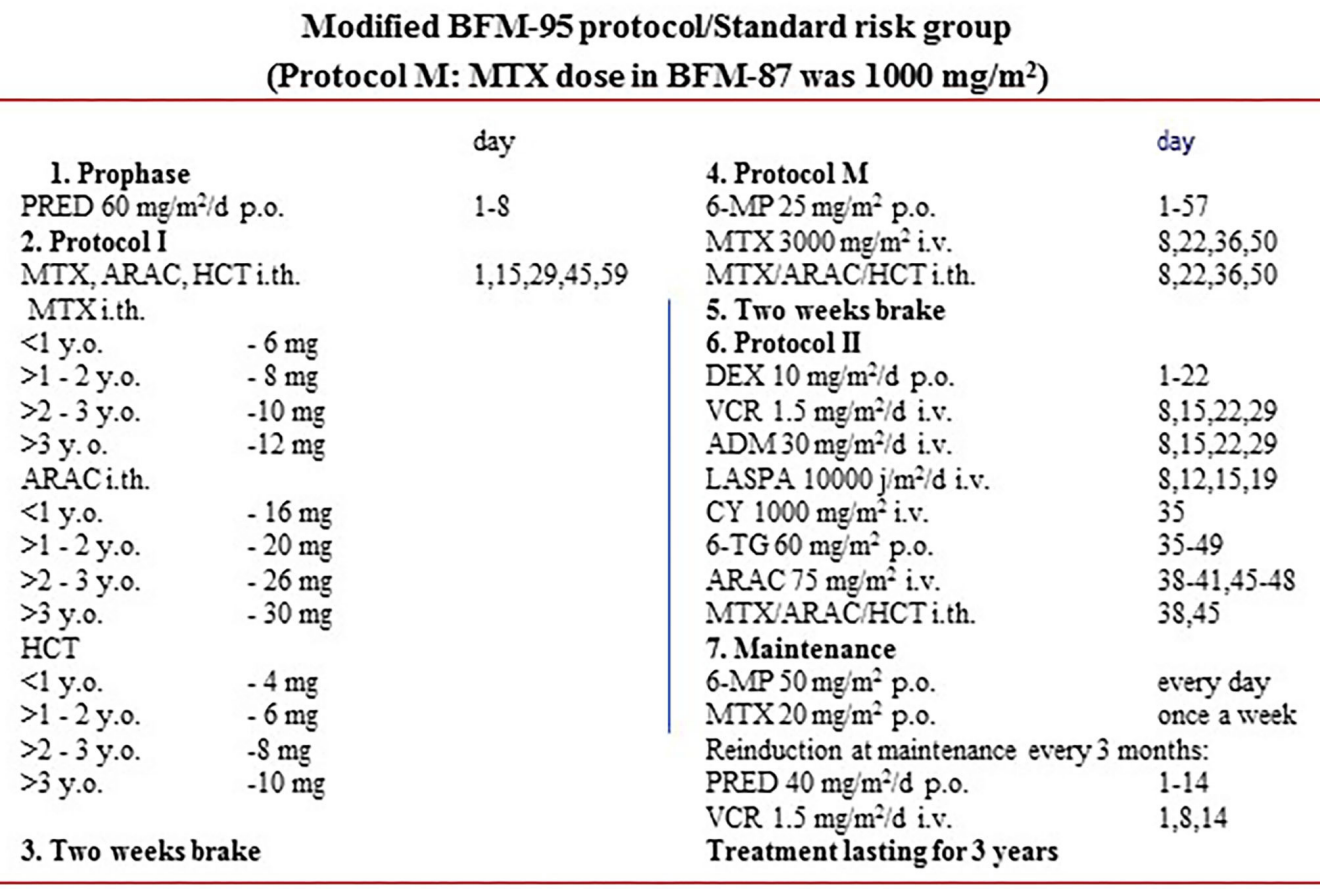
Schema of modified BFM95/87 protocols.

**Figure 2:**
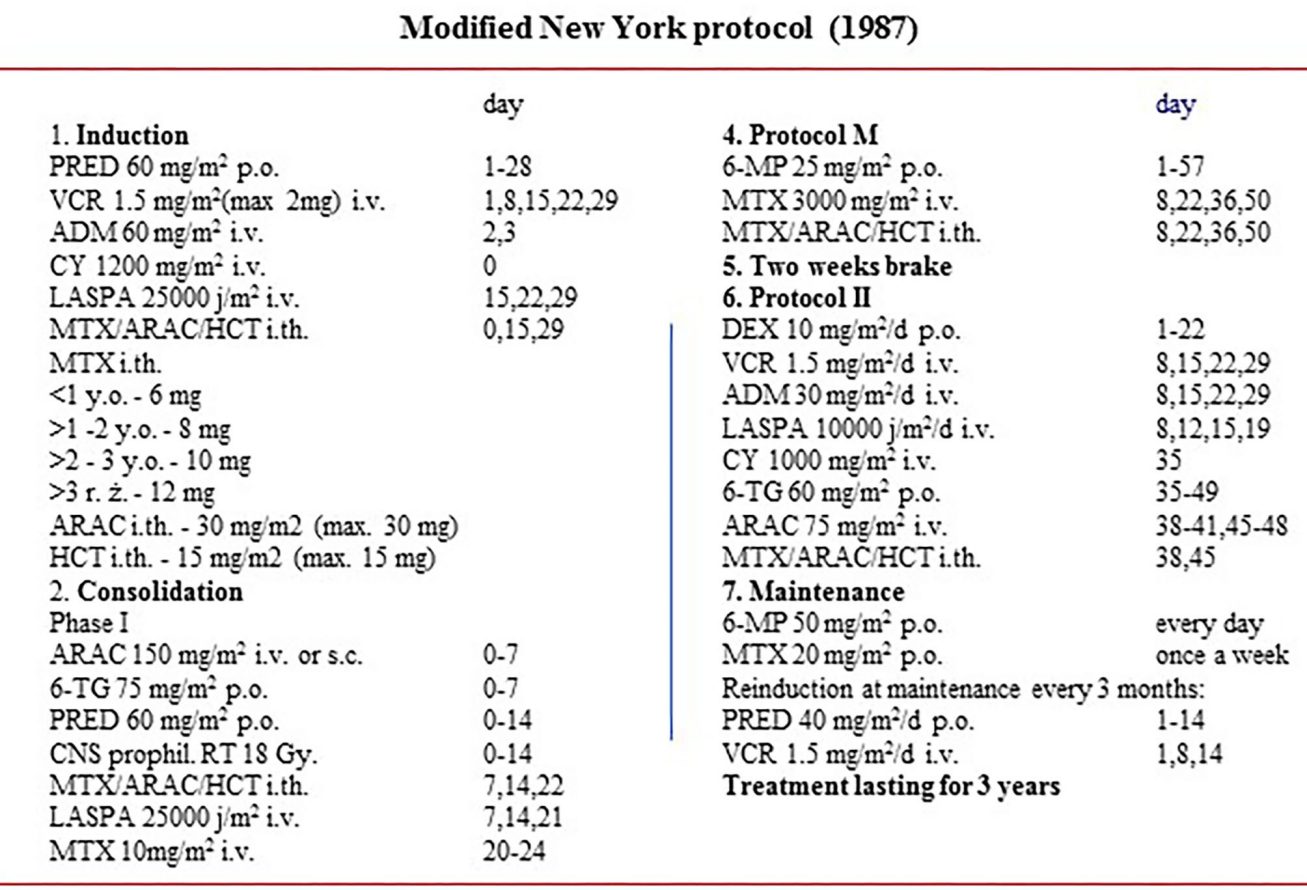
Schema of modified New York protocol.

Subjects were divided into three groups depending on treatment protocols (Figures 1, 2). The first group (*n* = 45, 29 boys, 64.4%) received treatment according to modified New York protocols (NY). Participants were aged from 1.9 to 18 years at diagnosis and from 4.9 to 26.4 years at the time of a follow-up examination.

The second group of 64 children (31 boys, 48.4%) was treated with previously modified BFM protocols (pBFM): 10 patients treated with BFM81, 83, and 86; and 54 patients treated with BFM87. Similarly as in NY, preventive and/or therapeutic central nervous system radiotherapy was performed according to specific protocols. The average age of the participants was 4.4 ± 2.8 (1–14.8) years and 18.3 ± 4.1 (11.3–28.4) years at the onset of treatment and a follow-up examination, respectively.

The third group of 106 children (48 boys, 45.3%) received treatment according to the BFM95 protocol without cranial radiotherapy. Within the group, two patients were given second-line chemotherapy due to relapse. The average age of the participants at the onset and a follow-up examination was 5.3 ± 3.1 (mean: 1–16.3) years and 11.5 ± 4.3 (mean: 5–24.4) years, respectively.

Central nervous system involvement localization was detected in seven children: one in NY, one in BFM95, and five cases in the pBFM.

Cumulative vincristine doses in NY fluctuated from 26 to 89 mg/m^2^ (mean: 60.8 mg/m^2^) and in BFM was 30 mg/m^2^. Two patients with ALL relapses received cumulative vincristine dose of 35 mg/m^2^. Radiotherapy dose was 13–36.4 (mean: 18.4) Gy in pBFM and 18.2–24 (mean: 18.3) Gy in NY, respectively.

At the follow-up visits, all patients underwent a standardized neurological examination with a detailed assessment of motor and sensory pathways.

The control group consisted of 72 patients (42 males, 58.3%) aged from 2 to 23 years with a mean of 10.4 ± 5.7 years who were hospitalized at the Department of Neurology due to a headache, first-time seizures, behavioral disorders, or neurotic disorders. The history and physical examination did not indicate peripheral nerves or muscle disorders. The results achieved from the control group were used to create customized reference values in our study.

### ENG and EMG Methodology

All patients underwent ENG examination of sensory and motor fibers of at least two long nerves, as well as quantitative EMG of at least one distal muscle and resting electromyographic record from two additional arbitrary muscles (one distal and one proximal). All neurophysiological tests were performed with Keypoint (MedtronicDantec) and described by one licensed physician.

ENG was performed using stimulating and recording superficial electrodes, and EMG with disposable standard concentric electrodes. To avoid the negative effect of a low temperature on conduction parameters, the skin temperature was measured before the test using a surface thermometer and, if necessary, the tested limb was heated to at least 34°C. The motor fibers of the peroneal nerve and sensory fibers of the sural nerve of the lower limb were selected for stimulation. The motor and sensory fibers of the median nerve were examined in the upper limb. The motor fibers of the median nerve were stimulated in typical places—over the wrist, at the antecubital fossa, and shoulder—and the recording electrode was located over the thenar. The peroneal nerve was stimulated distally over the tarsus, behind the fibula head, and below the popliteal area. The recording electrode was placed over the extensor digitorum brevis.

Recording of sensory response parameters from the sural nerve was done. The superficial electrode was located behind the lateral ankle, stimulating the nerve in one-third of the lower posterior surface of the calf slightly laterally from the midline, whereas the sensory potential from the median nerve was recorded at the site of motor nerve fibers' distal stimulation.

Sensory fibers were stimulated on the skin of the palmar surface of the forefinger proximal phalanx.

Selected electroneurographic parameters were analyzed in detail: standardized distal latency (dLat), motor nerve conduction velocity (MCV), compound motor action potential (CMAP), as well as the presence and the latency of the F wave. Sensory nerve action potential (SNAP) parameters were also evaluated: the latency (Lat), sensory nerve conduction velocity (SCV), and its amplitude. Moreover, standardized latency was calculated for each patient to avoid the inaccuracy resulting from the inability to comply with the standard location of stimulation and recording electrodes. The standardization was performed by calculating the quotient of the obtained latency value per 1 cm of distance between the stimulating and recording electrodes.

The anterior tibial and the vastus lateralis muscles in the lower limb as well as the interosseous muscle I in the upper limb were examined in EMG. The selected parameters of motor unit action potentials (MUAP) were assessed: their duration, amplitude, area, and polyphasia.

The study protocol complied with the Declaration of Helsinki and was approved by the local Ethics Committee (Consent No. KBET/131/B/207). All parents as well as adolescent and adult patients signed written informed consent before joining the study.

### Statistical Analysis

Statistical analyses were performed with Statistica 12.0 (StatSoft, Statistica 12.0, Tulsa, Oklahoma, USA) software. Continuous variables are expressed as mean ± SD and categorical variables as number (percentage). Continuous variables were first checked for normal distribution by the Shapiro–Wilk statistic. Differences among the two groups were compared by Student's *t*-test when normally distributed or by the Mann–Whitney *U*-test with a test for non-normally distributed variables. The differences among the three groups were compared using the ANOVA test when normally distributed or by the Kruskal–Wallis test with a test for multiple comparisons for non-normally distributed variables. Categorical variables were analyzed by the χ^2^ test and Fisher's exact test depending on the size of the analyzed groups. *P*-value of < 0.05 was considered statistically significant.

## Results

### Clinical Profile and Abnormalities Is Neurological Examination

No significant difference in terms of gender distribution in ALL patients and the control group was observed (male gender: 50.2 vs. 58.3%, *p* = 0.234). However, the comparison of patients treated with particular protocols revealed differences in gender (*p* = 0.047), the age of the onset of oncological treatment (*p* = 0.011), and the age of the control (*p* < 0.001) (Table 1). There were remarkably more male patients among the NY group than in BFM95 (64.4 vs. 45.3%, *p* = 0.041). NY patients were the oldest at the beginning of the anticancer treatment, whereas the patients from the pBFM group were the youngest (NY vs. pBFM, *p* = 0.011). However, pBFM patients were the oldest at the time of a follow-up examination.

**Table 1:** Clinical characteristics and identified neurological symptoms in patients in the study groups.

	**NY**	**pBFM**	**BFM95**	***P-*value**
Male gender	29 (64.4)	31 (48.4)	48 (45.3)	0.047^*^
Starting treatment, years	7.1 ± 4.8	4.4 ± 2.8	5.3 ± 3.1	0.011^**^
Age of control, years	14.4 ± 5.4	18.3 ± 4.1	11.5 ± 4.3	<0.001
Polyneuropathy—treatment	26 (57.8)	23 (35.9)	53 (50.0)	0.145^***^
Polyneuropathy—control	31 (68.9)	20 (32.2)	60 (58.0)	<0.001^****^
Average muscle strength in the Lovett scale	4.79	4.94	4.93	0.045^*****^
Reduced muscle strength	10 (22.2)	4 (6.5)	12 (11.5)	0.013^∧^
Hypo/areflexia	28 (62.2)	19 (30.7)	59 (56.2)	0.001^∧∧^
Muscle atrophy	7 (15.6)	2 (3.2)	2 (1.9)	0.034^∧∧∧^

* *NY vs. BFM95 p = 0.041*.

** *NY vs. pBFM p = 0.011*.

*** *NY vs. pBFM p = 0.024*.

**** *NY vs. pBFM p < 0.001, pBFM vs. BFM95 p = 0.002*.

***** *NY vs. pBFM p = 0.031*.

∧ *NY vs. pBFM p = 0.017*.

∧∧ *NY vs. pBFM p = 0.001, pBFM vs. BFM95 p = 0.001*.

∧∧∧ *NY vs. pBFM p = 0.023, NY vs. BFM95 p = 0.001*.

*BFM, Berlin–Frankfurt–Münster protocol; NY, New York protocol*.

Clinical symptoms of polyneuropathy were noted among 102 (47.4%) children at the ALL therapy and 111 (51.6%) patients at the second examination. Polyneuropathy at the time of treatment was diagnosed in 57.8% participants from NY group, 35.9%—pBFM and 50.0%—BFM95 (*p* = 0.145). The significantly highest incidence of polyneuropathy upon the second examination was observed in the NY group (*p* < 0.001). In direct comparison, the incidence was higher than in both remaining protocols (*p* < 0.001 vs. pBFM, *p* = 0.002 vs. BFM95).

Neurological examinations were performed each time before the electroneurographic testing. Significant differences between therapeutic protocols were observed in average muscle strength graded in Lovett scale (*p* = 0.045) and in the frequency of noticed reduced muscle strength (*p* = 0.013) as well as in the absence of deep tendon reflexes (*p* = 0.001) and muscle atrophy (*p* = 0.034; Table 1). The negative impact of the oncological treatment on muscle strength was particularly noticeable among NY patients. Abnormalities in deep tendon reflexes were most frequently found in the neurological examination (in 62.2% of NY patients, 30.7%—pBFM and 56.2%—BFM95). Muscle atrophy was observed in 15.6% of NY patients and its frequency was significantly higher in comparison with both remaining therapeutic protocols (*p* = 0.023 vs. pBFM, *p* = 0.001 vs. BFM95). None of the patients showed signs of impaired superficial and deep perception.

### Electroneurographic Examination

Features of registered electrophysiological parameters were analyzed in detail. The results remained within normal ranges in 15.6% of NY patients, 12.5% of pBFM, and in 13.2% of BFM95. In all of the treatment protocols, abnormalities of motor-sensory neuropathy have dominated (NY: 62.2%, pBFM: 40.6%, BFM95: 51.9%), whereas the isolated sensory neuropathy was the least often registered (NY: 4.4%, pBFM: 15.6%, BFM95: 8.5%). The distribution of particular neuropathy types did not differ significantly (*p* = 0.219). Neurophysiological abnormalities did not differ either (*p* = 0.360). The most common abnormality within all the protocols was demyelination (NY: 44.4%, pBFM: 59.4%, BFM95: 41.5%), in contrast to the least frequently registered isolated axonal changes (NY: 4.4%, pBFM: 4.7%, BFM95: 8.5%).

#### ENG—Motor Conduction in Median and Peroneal Nerves

Median standardized dLat registered during stimulation of the median nerve was significantly prolonged in ALL survivors in comparison with the control group (0.61 vs. 0.49 ms/cm, *p* < 0.001). Simultaneously, no significant impact of oncological treatment in the remaining parameters was detected. Prolonged dLat was the most commonly identified abnormality (Table 2). Criteria of prolonged dLat were fulfilled in 67.4% of NY patients, 31.3% of pBFM, and 49.1% of BFM95. The difference in a direct comparison between protocols was statistically significant (*p* = 0.001). Moreover, dLat was significantly prolonged in NY patients in comparison with the control group (0.65 vs. 0.49 ms/cm, *p* = 0.004).

**Table 2:** Conduction parameters in the study groups.

**Motor nerves**		**NY**	**pBFM**	**BFM95**	**Control**	***P-*value**
Median nerve	Prolonged dLat	31 (67.4)	20 (31.3)	52 (49.1)	–	0.001
	Reduced amplitude CMAP	9 (19.6)	7 (10.9)	19 (17.9)	–	0.383
	Reduced MCV	8 (17.4)	3 (4.7)	10 (9.4)	–	0.085
	Prolonged F-wave latency	8 (17.4)	4 (6.3)	10 (9.4)	–	0.153
	dLat, ms/cm	0.65	0.61	0.58	0.49	0.004^*^
	Amplitude, mV	9.54	10.04	9.08	9.33	0.228
	MCV, m/s	57.76	59.96	58.91	57.62	0.282
	F-wave latency, ms	19.77	19.95	19.43	19.68	0.673
Peroneal nerve	Prolonged dLat	17 (37.8)	24 (37.5)	44 (41.5)	–	0.843
	Reduced amplitude CMAP	9 (20.0)	5 (7.8)	27 (25.5)	–	0.018
	Reduced MCV	8 (17.8)	4 (6.3)	10 (9.4)	–	0.138
	Prolonged F-wave latency	17 (37.8)	12 (18.8)	19 (17.9)	–	0.020
	dLat, ms/cm	0.56	0.57	0.56	0.48	0.027^**^
	Amplitude, mV	4.11	5.34	4.12	5.14	<0.001^***^
	MCV, m/s	50.42	51.91	52.73	51.43	0.080
	F-wave latency, ms	30.03	34.32	31.54	29.82	0.024
**Sensory nerves**		**NY**	**pBFM**	**BFM95**	**Control**	***P*****-value**
Median nerve	Prolonged Lat	19 (42.2)	13 (20.6)	35 (33.0)	–	0.050
	Reduced amplitude SNAP	3 (6.7)	7 (11.1)	4 (3.8)	–	0.147
	Reduced SCV	19 (42.2)	15 (23.8)	37 (34.9)	–	0.117
	Lat, ms/cm	0.25	0.24	0.25	0.23	0.053
	Amplitude, mV	15.42	20.46	20.55	26.82	<0.001^*^
	SCV, m/s	53.31	55.92	52.84	57.82	<0.001^**^
Sural nerve	Prolonged Lat	8 (17.8)	6 (9.5)	21 (20.0)	–	0.200
	Reduced amplitude SNAP	14 (31.1)	9 (14.3)	18 (17.1)	–	0.068
	Reduced SCV	7 (15.6)	9 (14.3)	12 (11.4)	–	0.751
	Lat, ms/cm	0.25	0.25	0.24	0.24	0.694
	Amplitude, mV	18.89	22.47	22.64	26.00	0.019^***^
	SCV, m/s	55.72	55.11	55.81	55.85	0.923

*Motor conduction parameters in the studied groups*:

* *NY vs. control p = 0.004*.

** *pBFM vs. control p = 0.051*.

*** *NY vs. pBFM p = 0.025, pBFM vs. BFM95 p = 0.005*.

Sensory conduction parameters in the studied groups:

* *NY vs. control p < 0.001*.

#x0002A;*: *NY vs. control p = 0.046, pBFM vs. BFM95 p = 0.024, BFM95 vs. control p = 0.020*.

*** *NY vs. control p = 0.008*.

*BFM, Berlin–Frankfurt–Münster protocol; NY, New York protocol; dLat, standardized distal latency; CMAP, compound motor action potential; MCV, motor nerve conduction velocity; Lat, standardized terminal latency; SNAP, sensory nerve action potential; SCV, sensory nerve conduction velocity*.

Similar abnormalities were also found in the peroneal nerve. Prolonged dLat was most commonly observed and its values were significantly prolonged in ALL survivors in comparison with the control group (0.56 vs. 0.48 ms/cm, *p* < 0.001). However, no differences between particular protocols were found (Table 2). That difference was noticed in the frequency of reduced CMAP amplitude (*p* = 0.018), which was registered in 20.0% of NY patients, 7.8% of pBFM, and in 25.5% of BFM95, and in the prolongation of F-wave latency (*p* = 0.020). Prolongation of F-wave latency was observed in 37.8% of NY patients.

The values of particular motor conduction parameters in the peroneal nerve were notably different between analyzed groups in terms of dLat (*p* = 0.027), potentials amplitude (*p* < 0.001), and F-wave latency (*p* = 0.024). Borderline level of significance for difference in dLat between pBFM protocol and the control group was demonstrated (0.57 vs. 0.48 ms/cm, *p* = 0.051). The value of potentials amplitude was significantly higher in the pBFM group (5.34 mV) in comparison with NY (4.11 mV, *p* = 0.025) and BFM95 (4.12 mV, *p* = 0.005).

#### ENG—Sensory Conduction in Median and Sural Nerves

Comparing average conduction parameters in sensory fibers of the median nerve, notable differences within all parameters between ALL survivors and the control group were detected. ALL survivors had significantly prolonged latency of registered potentials (0.24 vs. 0.23 ms/cm, *p* = 0.005), decreased amplitude (19.40 vs. 26.79 mV, *p* = 0.043), and decreased SCV (53.83 vs. 57.82 m/s, *p* = 0.011). Prolonged Lat and reduced SCV were the most frequent abnormalities within all analyzed protocols although with no relevant differences in terms of its frequency in direct inter-group comparison (Table 2). Such variations were demonstrated in terms of the value of potentials amplitude (*p* < 0.001) and SCV (*p* < 0.001). The value of amplitude in the NY group was the lowest among all analyzed groups. That value was significantly lower than in the control group (15.42 vs. 26.82 mV, *p* < 0.001). Complex differences between analyzed groups were observed in terms of SCV. The lowest values, which were confirmed by conducted subanalyses, were registered in NY and BFM95 patients (Table 2).

A similar pattern of described abnormalities was also noticed among potentials registered in the lower extremity. Comparing sural nerve parameters of ALL survivors and control, a significant influence of oncological treatment on reduction of amplitude potentials (21.80 vs. 26.00 mV, *p* = 0.035) was observed. Similarly, as in sensory conduction in median nerve, no difference in frequency of particular abnormalities in analyzed protocols was found. Equally as in the case of the median nerve, the registered amplitude was the lowest within the NY group and the values were remarkably lower than in the control group (18.89 vs. 26.00 mV, *p* = 0.008).

#### EMG—Motor Unit Action Potential Parameters in Tibialis Anterior, Vastus Lateralis, and Interosseous I Muscles

In the tibialis anterior muscle, ALL patients showed a significant difference in the individual MUAP parameters compared with the control group. Increased amplitude (945.43 vs. 536.41 μV, *p* < 0.001), prolonged duration (11.22 vs. 9.17 ms, *p* < 0.001), and increased area (1,481.38 vs. 728.58 μV·s, *p* < 0.001) of recorded potentials in ALL survivors were observed. MUAP were the most severely disturbed in the NY patients, and the differences were significant. The most subtle changes were observed in the BFM95 group. The smallest percentage of polyphase potentials was registered in the pBFM group (Table 3). Amplitude, duration, and area values were significantly increased in the NY group compared with the control group as well as to pBFM and BFM95. Moreover, the registered potentials were also significantly prolonged in a direct comparison of pBFM and BFM95 with the control group (Table 3).

**Table 3:** Motor unit action potential parameters in the studied groups.

		**NY**	**pBFM**	**BFM95**	**Control**	***P-*value**
Tibialis anterior muscle	Increased amplitude of MUAP	31 (68.9)	14 (22.2)	22 (20.8)	–	<0.001
	Prolonged MUAP	36 (80.0)	37 (58.7)	66 (62.3)	–	0.039
	Increased area of MUAP	27 (60.0)	7 (11.1)	9 (8.5)	–	<0.001
	Polyphasia	27 (60.0)	33 (52.4)	66 (62.3)	–	0.406
	Amplitude, μV	1440.72	856.13	786.62	536.41	<0.001^*^
	Duration, ms	12.73	11.02	10.62	9.11	<0.001^**^
	Area, μV·s	2,509.86	1,278.02	1,162.57	728.55	<0.001^***^
	Phases	15.72	12.51	15.45	13.15	0.200
Vastus lateralis muscle	Increased amplitude of MUAP	26 (61.9)	28 (45.9)	19 (19.2)	–	<0.001
	Prolonged MUAP	16 (35.7)	8 (13.1)	13 (13.1)	–	0.001
	Increased area of MUAP	22 (52.4)	10 (16.4)	8 (8.1)	–	<0.001
	Polyphasia	19 (45.2)	28 (45.9)	32 (32.3)	–	0.137
	Amplitude, μV	1177.12	898.22	841.32	579.05	0.005^****^
	Duration, ms	12.43	11.84	11.14	9.41	<0.001^*****^
	Area, μV·s	1,949.52	1,369.13	1,147.92	719.16	<0.001^∧^
	Phases	11.92	10.32	7.95	6.90	0.028
Interosseous muscle I	Increased amplitude of MUAP	21 (46.7)	11 (17.7)	10 (9.9)	–	<0.001
	Prolonged MUAP	13 (28.9)	6 (9.7)	8 (7.9)	–	0.001
	Increased area of MUAP	17 (35.6)	3 (4.8)	4 (4.0)	–	<0.001
	Polyphasia	27 (60.0)	24 (38.7)	50 (49.5)	–	0.038
	Amplitude, μV	1,326.92	875.31	757.26	575.02	<0.001^∧∧^
	Duration, ms	10.32	9.22	8.71	8.21	<0.001^∧∧∧^
	Area, μV*s	1,934.71	1,104.07	910.33	664.02	<0.001^∧∧∧∧^
	Phases	15.71	11.48	12.21	18.04	0.072

* *NY vs. pBFM p < 0.001, NY vs. BFM95 p < 0.001, NY vs. control p < 0.001*.

** *NY vs. pBFM p < 0.001, NY vs. BFM95 p < 0.001, NY vs. control p < 0.001, pBFM vs. control p = 0.001, BFM95 vs. control p = 0.013*.

*** *NY vs. pBFM p < 0.001, NY vs. BFM95 p < 0.001, NY vs. control p < 0.001*.

**** *NY vs. BFM95 p = 0.0368, NY vs. control p < 0.001*.

***** *NY vs. BFM95 p = 0.004, NY vs. control p = 0.004, pBFM vs. control p = 0.037*.

∧ *NY vs. pBFM p < 0.001, NY vs. BFM95 p < 0.001, NY vs. control p < 0.001*.

∧∧ *NY vs. pBFM p < 0.001, NY vs. BFM95 p < 0.001, NY vs. control p = 0.019*.

∧∧∧ *NY vs. pBFM p = 0.036, NY vs. BFM95 p = 0.001*.

∧∧∧∧ *NY vs. pBFM p < 0.001, NY vs. BFM95 p < 0.001, NY vs. control p = 0.027*.

*BFM, Berlin–Frankfurt–Münster protocol; NY, New York protocol; MUAP, motor unit action potential*.

Findings described earlier were also confirmed in other examined muscles. Potentials recorded in vastus lateralis and interosseous I muscles also showed significant changes in ALL survivors. In vastus lateralis muscle, significant differences were found in the amplitude (928.31 vs. 579.04 μV, *p* < 0.001), duration (11.65 vs. 9.44 ms, *p* = 0.013), and MUAP area (1,381.43 vs. 719.16 μV·s, *p* < 0.001) between ALL survivors and the control group. In addition, in interosseous I muscle, amplitude (909.63 vs. 575.05 μV, *p* < 0.001) and MUAP area (1,178.83 vs. 644.02 μV·s, *p* < 0.001) were significantly increased in patients with ALL. However, the prolonged duration of MUAP in that muscle did not differ significantly from the control group (9.22 vs. 8.21 ms, *p* = 0.173). Once again, the NY group presented the most severe neuropathic changes and BFM95—the mildest (Table 3).

### The Impact of Radiotherapy on the Electrophysiological Parameters

To assess the impact of radiotherapy on the shape of recorded neurophysiological responses, a joint analysis of NY and pBFM groups (treated with radiotherapy) was performed. In the groups that differed in terms of radiotherapy, the gender distribution and mean age at the onset of the treatment were similar, while patients with radiotherapy were significantly older than those without radiotherapy (RT) at the time of the second examination (*p* < 0.001; Table 4). No increase in the incidence of polyneuropathy was observed in irradiated patients at ALL therapy and follow-up. The differences in the frequency of strength impairment, weakness, or absence of deep tendon reflexes as well as muscular atrophy were statistically insignificant in these two groups. The mean muscular strength in patients treated with RT was also slightly lower than in the BFM95 group, but the difference did not reach statistical significance (Table 4).

**Table 4:** Clinical characteristics and identified neurological symptoms in irradiated and non-irradiated groups.

	**NY + pBFM**	**BFM95**	***P-*value**
Male gender	60 (55.1)	48 (45.3)	0.196
Starting treatment, years	5.4 ± 3.2	5.3 ± 3.3	0.755
Age of control, years	16.7 ± 4.1	11.5 ± 4.2	<0.001
Polyneuropathy—treatment	49 (55.1)	53 (57.0)	0.793
Polyneuropathy—control	51 (47.7)	60 (57.7)	0.144
Average muscle strength in the Lovett scale	4.88	4.93	0.757
Reduced muscle strength	14 (13.1)	12 (11.5)	0.733
Hypo/areflexia	40 (43.8)	59 (56.2)	0.074
Muscle atrophy	7 (6.5)	2 (1.9)	0.085

*BFM, Berlin–Frankfurt–Münster protocol; NY, New York protocol*.

No statistically significant differences in the frequency of changes in motor conduction parameters in the median nerve were found in the irradiated group. Statistically significant differences were also not shown in the particular analyzed conductivity parameters (Table 5).On the other hand, motor conductivity in the peroneal nerve, CMAP amplitude reduction (13.0 vs. 25.5%, *p* = 0.019), higher amplitude values (4.82 vs. 4.13 mV, *p* = 0.014), and lower MCV (51.29 vs. 52.65 m/s, *p* = 0.047) were observed in the irradiated group (Table 5).

**Table 5:** Conduction parameters in irradiated and non-irradiated groups.

**Motor nerves**	**NY + pBFM**	**BFM95**	***P-*value**	
Median nerve	Prolonged dLat	51 (46.8)	52 (49.1)	0.739
	Reduced amplitude CMAP	16 (14.7)	19 (17.9)	0.519
	Reduced MCV	11 (10.2)	10 (9.4)	0.853
	Prolonged F-wave latency	12 (11.5)	10 (9.8)	0.687
	dLat, ms/cm	0.63	0.58	0.157
	Amplitude, mV	9.88	9.08	0.067
	MCV, m/s	59.07	58.91	0.874
	F-wave latency, ms	19.88	19.43	0.226
Peroneal nerve	Prolonged dLat	41 (38.0)	44 (41.5)	0.596
	Reduced amplitude CMAP	14 (13.0)	27 (25.5)	0.019
	Reduced MCV	12 (11.3)	10 (9.4)	0.652
	Prolonged F-wave latency	29 (28.7)	19 (18.3)	0.077
	dLat, ms/cm	0.56	0.56	0.886
	Amplitude, mV	4.82	4.13	0.014
	MCV, m/s	51.29	52.65	0.047
	F-wave latency, ms	32.68	31.46	0.301
**Sensory nerves**	**NY** **+** **pBFM**	**BFM95**	***P-*****value**	
Median nerve	Prolonged Lat	32 (29.6)	35 (33.0)	0.593
	Reduced amplitude SNAP	10 (9.3)	4 (3.8)	0.099
	Reduced SCV	34 (31.5)	37 (34.9)	0.595
	Lat, ms/cm	0.24	0.25	0.434
	Amplitude, mV	18.31	20.51	0.060
	SCV, m/s	54.80	52.84	0.016
Sural nerve	Prolonged Lat	14 (13.0)	21 (10.0)	0.165
	Reduced amplitude SNAP	23 (21.3)	18 (17.1)	0.441
	Reduced SCV	16 (14.8)	12 (11.4)	0.464
	Lat, ms/cm	0.25	0.25	0.570
	Amplitude, mV	20.98	22.64	0.238
	SCV, m/s	55.33	55.82	0.627

*BFM, Berlin–Frankfurt–Münster protocol; NY, New York protocol; dLat, standardized distal latency; CMAP, compound motor action potential; MCV, motor nerve conduction velocity; Lat, standardized terminal latency; SNAP, sensory nerve action potential; SCV, sensory nerve conduction velocity*.

In terms of sensory conduction in the median nerve, no significant differences in the frequency of changes in conduction parameters were demonstrated in the compared groups. SCV, although normal, was significantly slower in the BFM95 group compared to those treated with radiotherapy (54.80 vs. 52.84 m/s, *p* = 0.016; Table 5). There were also no additional differences in the sensory conduction in the sural nerve (Table 5).

The most marked effect of radiotherapy on electrophysiological parameters was noted in EMG. In patients treated with NY and pBFM protocols, a general negative effect of radiation on recorded MUAP parameters of the lower limb (tibialis anterior and vastus lateralis) and upper limb (interosseous I) corresponding to more severe neuropathic changes was observed. In the case of protocols containing radiotherapy, higher values of amplitude, duration, and MUAP area were found in all examined muscles (Table 6). Moreover, a higher incidence of polyphasia (45.6 vs. 32.3%, *p* = 0.046) and higher values of recorded phases (10.95 vs. 7.94, *p* = 0.007) were found in vastus lateralis muscle (Table 6).

**Table 6:** Motor unit action potential parameters in irradiated and non-irradiated groups.

		**NY + pBFM**	**BFM95**	***P-*value**
Tibialis anterior muscle	Increased amplitude of MUAP	45 (41.7)	22 (20.8)	<0.001
	Prolonged MUAP	73 (68.0)	66 (62.3)	0.249
	Increased area of MUAP	34 (31.5)	9 (8.5)	<0.001
	Polyphasia	60 (55.6)	66 (62.3)	0.278
	Amplitude, μV	1099.65	786.65	<0.001
	Duration, ms	11.69	10.61	<0.001
	Area, μV·s	1791.28	1162.54	<0.001
	Phases	13.83	15.44	0.238
Vastus lateralis muscle	Increased amplitude of MUAP	54 (52.4)	19 (19.2)	<0.001
	Prolonged MUAP	24 (23.3)	13 (13.1)	0.057
	Increased area of MUAP	32 (31.1)	8 (8.1)	<0.001
	Polyphasia	47 (45.6)	32 (32.3)	0.046
	Amplitude, μV	1011.93	841.33	0.044
	Duration, ms	12.05	11.13	<0.001
	Area, μV·s	1605.79	1147.88	<0.001
	Phases	10.95	7.94	0.007
Interosseous muscle I	Increased amplitude of MUAP	32 (29.9)	10 (9.9)	<0.001
	Prolonged MUAP	19 (17.8)	8 (7.9)	0.033
	Increased area of MUAP	20 (18.7)	4 (4.0)	0.001
	Polyphasia	51 (47.7)	50 (49.5)	0.845
	Amplitude, μV	1057.69	757.18	<0.001
	Duration, ms	9.68	8.74	<0.001
	Area, μV·s	1439.49	910.29	<0.001
	Phases	13.13	12.23	0.508

*BFM, Berlin–Frankfurt–Münster protocol; NY, New York protocol; MUAP, motor unit action potential*.

## Discussion

To our knowledge, the current study is the largest and one of the most comprehensive ones among those examining neurophysiological disturbances in ENG and EMG in the group of long-term childhood ALL survivors. Moreover, as demonstrated for the first time, radiotherapy, which was more often present in therapeutic protocols in the past, can also have a negative effect on peripheral nerve conduction parameters.

In our study, the majority of analyzed patients experienced generalized motor-sensory neuropathy. Moreover, depending on the analyzed segment of the nervous system, both demyelination and axonal changes were found. Noteworthily, the analyzed group of patients varied in terms of time from completion of oncological treatment to conducting a neurophysiological examination, which in our study ranged from 0.3 to up to 20.9 years. An interesting review of the most important studies to date, which analyzed neurophysiological changes in ALL survivors, has recently been made by Bjornard et al. (2) in *Lancet*. As demonstrated, the four discussed studies were significantly diverse in terms of analyzed groups, follow-up time as well as observed neurophysiological changes. In addition, different neurophysiological assessment and reporting methods exclude the possibility of making direct comparisons and drawing definitive conclusions. In the largest study to date, published by Kandula et al. (11), a dominant role of sensory neuropathy was found in 50.5% of participants. The time from the completion of cancer treatment was similar to our study (1.5–29 years). However, the study was conducted on a diverse group of 121 patients with various childhood cancers, among whom patients with ALL accounted for ~52% of the subjects. Another interesting study is the report by Tay et al. (4) which has been based on a homogeneous group of 101 patients with ALL. It confirms our observations concerning the most important role of mixed motor-sensory neuropathy, which in this research was found in 68.3% of patients 4 years after the end of oncological treatment. The two remaining studies by Jain et al. (12) and Ramchandren et al. (13) were carried out on relatively small groups of ALL patients. Motor neuropathy dominated in both studies. In addition, in both studies, no control groups were included that would allow a relevant comparison of the obtained results.

In the analyzed NY, pBFM, and BFM95 therapeutic protocols, vincristine was the main chemotherapeutic agent responsible for the development of neurotoxicity. Since discovering its high efficacy in childhood ALL, vincristine has remained a standard component of the most commonly used therapeutic protocols (14). As described earlier, the mechanism of CIPN induction by vincristine is associated with the cessation of axonal transport by impaired microtubule function, which leads to axonal swelling and to nervous fiber damage (15). Other reports also pointed out the important role of mitochondria, whose impaired function may contribute to the development of oxidative stress (16) and disturbed Ca^2+^ homeostasis (17). Importantly, it has not unambiguously been established whether the other drugs used in polychemotherapy regimens, including the analyzed protocols, did not synergistically increase the neurotoxic effect of vincristine.

As we have shown in our study, also radiotherapy used in NY and pBFM protocols may play a potential role in polyneuropathy development. We reported significant similarity in motor and sensory conduction parameters between irradiated and non-irradiated patients. However, the effect of radiotherapy is apparently expressed in EMG analysis. The observed disturbances suggest the significant severity of denervation and neuropathic changes in irradiated patients. At the same time, it is worth emphasizing that in the majority of currently used ALL protocols, radiotherapy has been replaced by intrathecal chemotherapy due to a significant reduction of complications (18). Our study represents therefore a kind of “glance at the past therapeutic protocols.” There are several potential mechanisms that can explain our observations. Radiation-induced damage can be explained by the oxidation of the lipid bilayer (19), changes in microvascular permeability (20), and mitochondrial abnormalities inducing oxidative stress (21). The simultaneous increase of the CIPN by radiotherapy cannot be excluded as well.

Our study has several limitations. First, our study compared different protocols previously used in clinical practice. However, thanks to this, it is possible to study the impact of radiotherapy on the shape of registered neurophysiological potentials. Second, we did not use dedicated scales to assess the severity of observed neurotoxicity in our study. However, the objective analysis of the stated neuropathic changes with ENG and EMG was the main purpose of our study. Moreover, the reliability of these scales has been already undermined in the literature (22–24). Third, genetic methods, especially whole-exome sequencing, which are increasingly used to determine the individual predisposition to neurotoxicity development, have not been used (5, 25, 26).

## Conclusions

Detailed neurophysiological analysis in long-term childhood ALL survivors has shown generalized abnormalities in registered parameters with a dominant role of motor-sensory neuropathy. To our knowledge, the current study is the largest and one of the most comprehensive ones among those examining disturbances in ENG and EMG in this group of patients. Moreover, as demonstrated for the first time, radiotherapy can also have a negative effect on peripheral nerve conduction parameters expressed mainly in EMG.

## Data Availability Statement

The original contributions generated for the study are included in the article/supplementary material, further inquiries can be directed to the corresponding author/s.

## Ethics Statement

The studies involving human participants were reviewed and approved by Jagiellonian University Ethics Committee, Krakow, Poland (Consent No. KBET/131/B/207). All parents as well as adolescent and adult patients signed written informed consent before inclusion in the study.

## Author Contributions

SK, KoS, and SS contributed to the study concept and design. SK, IW-M, TK, EK, and AB performed diagnostic tests and collected relevant clinical data. KoS, PS, HT, and KlS conducted statistical analysis and wrote sections of the manuscript. SK and SS critically revised the article. All authors were responsible for the integrity and accuracy of the data and approved the submitted version.

## Conflict of Interest

The authors declare that the research was conducted in the absence of any commercial or financial relationships that could be construed as a potential conflict of interest.
